# Herpes Simplex Virus Proctitis Masquerading as Rectal Cancer

**DOI:** 10.3390/diseases7020036

**Published:** 2019-04-21

**Authors:** Folusakin Ayoade, Jose Armando Gonzales Zamora, Youley Tjendra

**Affiliations:** 1 Division of Infectious Diseases. Department of Medicine, Miller School of Medicine, University of Miami, Miami, FL 33136, USA; fxa375@med.miami.edu; 2 Department of Pathology and Laboratory Medicine, Miller School of Medicine, University of Miami, Miami, FL 33136, USA; youley.tjendra@jhsmiami.org

**Keywords:** herpes simplex virus, proctitis, rectal mass, rectal cancer, HIV

## Abstract

Herpes simplex virus (HSV) is the leading cause of proctitis in HIV-infected individuals. However, no cases of rectal masses secondary to HSV infection have been reported to date. Herein, we present the case of a 45-year-old man with HIV infection who developed rectal pain and bleeding, along with dysuria and voiding difficulty. Colonoscopy revealed proctitis and a rectal mass with features concerning for rectal cancer. Histologic sections of the rectal mass biopsy demonstrated colorectal mucosa with viral cytopathic changes, ulceration, granulation tissue, marked inflammatory infiltrate, and fibrinopurulent exudate. Immunohistochemistry for herpes simplex virus-1 was positive in epithelial cells demonstrating a viral cytopathic effect. The patient was treated with valacyclovir for 3 weeks, which led to complete resolution of his symptoms. Follow-up sigmoidoscopy at 6 months did not show any masses. Our case illustrates the importance of considering HSV in the differential diagnosis of rectal masses. We advocate the routine use of viral immunohistochemistry for the evaluation of rectal tumors, especially in patients with clinical manifestations and endoscopic findings consistent with proctitis.

## 1. Case Description

A 45-year-old man presented to the hospital with complaints of rectal pain and bright red blood with each bowel movement for the past 6 months. He also described cramping rectal pain alleviated by bowel movements and aggravated by sitting and straining. He denied fevers or chills, but endorsed 15-pound weight loss in the last 3 weeks due to anorexia and fear of having a painful bowel movement. He admitted to have receptive anal intercourse, but had not been sexually active for the past 14 months due to pain. He also complained of dysuria and straining to urinate with incomplete voiding. The patient had a history of HIV infection and treated syphilis 6 months ago. His antiretroviral treatment consisted of dolutegravir and darunavir/cobicistat. A physical exam revealed a soft, nontender, and nondistended abdomen with normal bowel sounds. A rectal exam showed normal rectal tone, tenderness to palpation, external hemorrhoids, and a deep anal fissure. Laboratory studies were significant for hemoglobin of 12.6 g/dL, a white blood cell count of 5.6 K/uL, and creatinine of 1.15 mg/dL. His CD4 count was 283 cells/uL and his HIV viral load was undetectable. Urinalysis was normal. Computed tomography revealed prominent concentric thickening of the mid and lower rectum with associated mesorectal fat stranding and lymphadenopathy, highly concerning for an underlying rectal neoplasm or severe proctocolitis.

**Figure 1:**
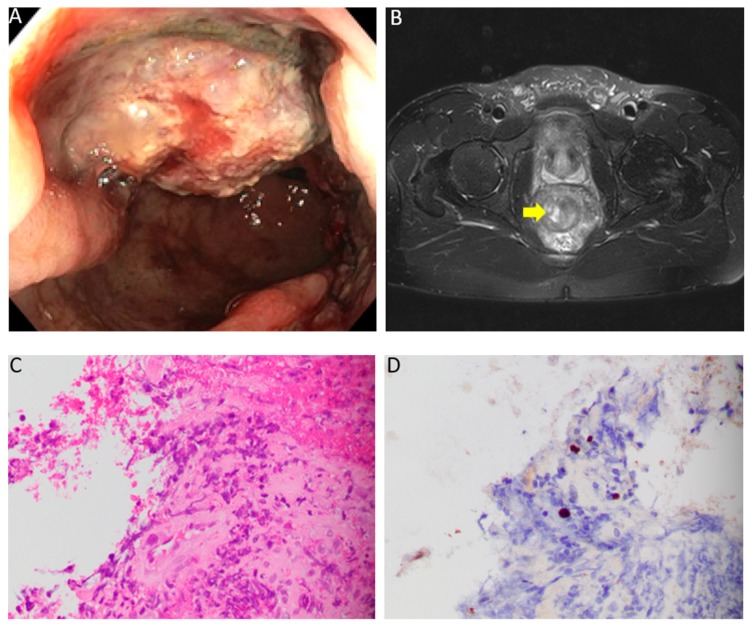
(**A**) Colonoscopy showing a fungating and infiltrative partially obstructing medium-sized mass in the rectum, 10–15 cm from the anal verge. (**B**) Axial T2-weighted MRI showing a polypoidal mass with a circumferential nodular border. More than 50% of the tumor had a very high T2 signal intensity compared to perirectal fat. (**C**) Histologic sections of the rectal mass biopsy demonstrate colorectal mucosa with ulceration, epithelial cells with nuclear inclusions, exuberant granulation tissue, marked lymphoplasmacytic and eosinophilic infiltrate, and fibrinopurulent exudate (Hematoxylin & Eosin, 400X). (**D**) Immunohistochemistry for herpes simplex virus-1 was positive in epithelial cells demonstrating a viral cytopathic effect (Herpes simplex virus-1 (HSV-1) immunohistochemistry, 400X).

For this reason, the patient underwent a colonoscopy that showed a fungating and infiltrative partially obstructing medium-sized mass in the rectum, 10–15 cm from the anal verge (Figure 1A). Discontinuous areas of nonbleeding ulcerated mucosa with no stigmata of recent bleeding were present in the rectum. Multiple biopsies were obtained. He was started empirically on doxycycline 100 mg every 12 hours for possible lymphogranuloma venereum or syphilis. Further infectious work-up revealed a negative gonorrhea/chlamydia PCR in urine and rectum. RPR (rapid plasma reagin) titer was 1:8; however, the patient had a titer of 1:32 6 months ago, consistent with history of adequately treated syphilis. Given the possibility of rectal cancer, magnetic resonance imaging (MRI) was ordered for staging, which demonstrated a polypoidal mass with a circumferential nodular border. More than 50% of the tumor had a very high T2 signal intensity compared with perirectal fat (Figure 1B). Lymphadenopathies of ≥9 mm were identified in the mesorectal/superior rectal space. Histologic sections of the biopsy revealed colorectal mucosa with ulceration, epithelial cells with viral nuclear inclusion, exuberant granulation tissue, marked lymphoplasmacytic and eosinophilic infiltrate, and fibrinopurulent exudate (Figure 1C). Immunohistochemistry for herpes simplex virus-1 was positive in epithelial cells demonstrating a viral cytopathic effect (Figure 1D), supporting the diagnosis of HSV infection. Immunostain for Cytomegalovirus (CMV) and Adenovirus were negative. Warthin–Starry stain was negative for spirochetes. Gomori methenamine silver (GMS) stain and trichrome stain failed to demonstrate fungal organisms or parasites. The overall histologic changes were not suggestive of human herpes virus-8 (HHV-8) or HPV infection; therefore, testing for these viruses was not performed. Doxycycline was discontinued and the patient was started on valacyclovir (1 g) every 8 hours for 21 days. At 3-month follow-up, the patient reported complete resolution of rectal pain and denied any further episodes of bloody stools. Three months later, a sigmoidoscopy was performed and revealed a normal rectal mucosa and no evidence of masses.

## 2. Discussion

The differential diagnosis of rectal masses in patients with HIV is broad and includes infectious causes, such as lymphogranuloma venereum, syphilis, CMV, and HHV-8 [1,2,3]. Non-infectious pathologies, such as lymphoma or squamous cell carcinoma, are also important entities to consider in this population [4,5]. We present a patient with herpes simplex virus (HSV) infection who was found to have a rectal mass, with an endoscopic appearance concerning for malignancy. This case constitutes the first report in the English literature of HSV manifesting as a rectal mass. Our patient also endorsed symptoms of proctitis, which is a clinical condition mostly associated with sexually transmitted infections, especially in men who have sex with men (MSM). Multiple infectious agents are known to cause proctitis. Gonorrhea, chlamydia, syphilis, and HSV are described as the most common [6]. In a recent review, HSV was identified as the leading cause of proctitis in patients with HIV infection [7]. There are two types of herpes simplex virus: HSV-1, which is typically associated with oropharyngeal infection, and HSV-2, which is mainly related to genital disease [8]. However, in recent years, HSV-1 has become a common cause of anogenital herpes in developed countries, probably secondary to increasing rates of orogenital sex practices, particularly in MSM [9,10]. HSV-1 was the etiologic agent in our patient.

Clinically, the individuals with proctitis report a great variety of symptoms that include rectal bleeding, pain, tenesmus, and diarrhea or constipation. The majority of patients affected by this condition share this spectrum of symptoms; however, HSV has unique manifestations that are relevant when evaluating a patient clinically. Most individuals with HSV proctitis present neurologic manifestations in the distribution of sacral roots. Difficulty in initiating micturition, posterior thigh pain or paresthesias of the buttock or perineal region, and impotence are neurologic complaints described by approximately 52% of men with this disease [11]. Besides rectal pain and bleeding, dysuria and voiding difficulty were also reported by our patient.

In terms of HSV diagnosis, it can be achieved by multiple techniques that include viral culture, PCR, and immunofluorescence staining. Among them, immunohistochemistry has proven to be one of the most useful tools, mainly in cases of fastidious or noncultivable micro-organisms. This technique is highly sensitive and specific, and is able to distinguish between numerous viruses (including HSV-1 and HSV-2) using specific monoclonal antibodies [12]. In our patient, the diagnosis of HSV infection was confirmed by immunohistochemistry using a mouse anti-human monoclonal antibody directed against herpes simplex virus-1 (Cell Marque Corporation, Rocklin, CA, concentration 0.15 ug/ml) on paraffin-embedded tissue sections. Regarding management, high doses of acyclovir or valacyclovir are often recommended in immunocompromised hosts [13]. Treatment with a prolonged course of valacyclovir in our patient led to complete resolution of symptoms and disappearance of the rectal mass.

## 3. Conclusions

Our case highlights the importance of considering HSV in the differential diagnosis of rectal masses, particularly in HIV-infected individuals with clinical manifestations and endoscopic findings consistent with proctitis. We also advocate the routine use of immunohistochemistry for viral pathogens as part of the diagnostic evaluation of rectal tumors.
